# Could the use of bedside lung ultrasound reduce the number of chest x-rays in the intensive care unit?

**DOI:** 10.1186/s12947-017-0113-8

**Published:** 2017-09-13

**Authors:** Etrusca Brogi, Elena Bignami, Anna Sidoti, Mohammed Shawar, Luna Gargani, Luigi Vetrugno, Giovanni Volpicelli, Francesco Forfori

**Affiliations:** 1 Department of Anesthesia and Intensive Care, University of Pisa, Pisa, Italy; 2 Department of Anesthesia and Intensive Care, IRCCS San Raffaele Scientific Institute, Via Olgettina 60, 20132 Milan, Italy; 3 Institute of Clinical Physiology - National Research Council, Pisa, Italy; 4 Department of Medicine, University of Udine, Udine, Italy; 5 Department of Emergency Medicine, San Luigi Gonzaga University Hospital, Orbassano, Torino, Italy

**Keywords:** Acute care, Ultrasound, Imaging, Ultrasound, X-Rays, Imaging, X-Rays

## Abstract

**Background:**

Lung ultrasound can be used as an alternative to chest radiography (CXR) for the diagnosis and follow-up of various lung diseases in the intensive care unit (ICU). Our aim was to evaluate the influence that introducing a routine daily use of lung ultrasound in critically ill patients may have on the number of CXRs and as a consequence, on medical costs and radiation exposure.

**Methods:**

Data were collected by conducting a retrospective evaluation of the medical records of adult patients who needed thoracic imaging and were admitted to our academic polyvalent ICU. We compared the number of CXRs and relative costs before and after the introduction of lung ultrasound in our ICU.

**Results:**

A total of 4134 medical records were collected from January 2010 to December 2014. We divided our population into two groups, before (Group A, 1869 patients) and after (Group B, 2265 patients) the introduction of a routine use of LUS in July 2012. Group A performed a higher number of CXRs compared to Group B (1810 vs 961, *P* = 0.012), at an average of 0.97 vs 0.42 exams per patient. The estimated reduction of costs between Groups A and B obtained after the introduction of LUS, was 57%. No statistically significant difference between the outcome parameters of the two groups was observed.

**Conclusions:**

Lung ultrasound was effective in reducing the number of CXRs and relative medical costs and radiation exposure in ICU, without affecting patient outcome.

## Background

Critically ill patients frequently need thoracic imaging due to the constant evolution of their clinical conditions. Computed tomography (CT) scans remain the gold standard imaging technique for thoracic evaluation, but transportation of patients outside the ICU is difficult and potentially harmful [1]. Chest CT scans expose patients to large doses of radiation and should be reserved for specific situations (e.g., the evaluation of mediastinal pathologies and confirmation of pulmonary embolism) [2–4]. Bedside chest X-ray (CXR) is still considered the standard of care for many diagnostic applications in the Intensive Care Unit (ICU). However, this imaging technique has important methodological limitations and often shows low accuracy [5]. Furthermore, it is important to consider radioprotection issues. Multiple radiologic imaging exams result in an increased incidence of radiation-induced cancer in the long-term [6].

Bedside sonography has become essential in the ICU for many common applications. Particularly, lung ultrasound (LUS) has been shown to be superior to CXR as a diagnostic tool for the diagnosis of some lung conditions in critically ill patients (i.e., pneumothorax, pleural effusion, consolidation, Ventilator-Associated-Pneumonia) [7–9]. Consequently, LUS may be considered a valid alternative to CXR in some specific situations. Potentially, a systematic application of LUS may be associated with a reduced use of routine CXR and chest CT scans, without affecting patient outcome while reducing radiation exposure [10]. Also, the use of bedside LUS could lead to reduced medical costs, as ultrasound scanners are relatively low-cost regarding maintenance and high durability compared to other imaging modalities [11].

In our ICU the routine use of LUS was introduced in July 2012: since then, we have performed a daily LUS round in our critically ill patients. The first aim of our study was to evaluate whether the routine daily use of LUS in our ICU influenced the number of diagnostic CXRs performed in our critically ill patients. The second aim was to estimate the effective reduction of medical costs and radiation exposure that was achieved by the introduction of LUS.

## Methods

This single-center, observational, retrospective cohort study took place in a university hospital in Italy from 2010 to 2014. We retrospectively analyzed our prospectively collected database. After approval by the Research Ethics Committee of Pisa (approval number 979, 07/04/2016), 4134 consecutive adult patients were enrolled in the study. Written informed consent was obtained from all the patients. This study adheres to the applicable STROBE guidelines. Data extraction was performed independently by two authors (S.M., S.A.) and any discrepancy resolved prior to final analysis by discussion with a third authors (B.E.).

We recruited adult patients admitted to our ICU from January 2010 to December 2014, who needed thoracic imaging during the ordinary clinical work-up. The inclusion criteria were as follows: age > 18 years, ability to provide written consent, and clinical indications for thoracic imaging test.

In the second half of 2012, we implemented ultrasound as the thoracic imaging technique of choice in our ICU. From then on, physicians could decide to use LUS instead of CXR for the first diagnosis, follow-up and monitoring of pleural-pulmonary conditions in the critically ill patients admitted to our ICU.

The cohort was divided into two groups using temporal criteria, as follows:

A. In **Group A**, we included patients admitted to the ICU from January 1, 2010 to May 31, 2012. During this period LUS was not yet implemented as a standard practice in our ICU and was only used sporadically on a consultancy basis. In this period, thoracic imaging was based on CXRs or thoracic CT scans as the standard of practice.
B. In **Group B**, we included patients admitted to the ICU from June 1, 2012 to December 31, 2014. In this period, thoracic ultrasound was introduced in our ICU and implemented as the standard of care for many applications: physicians could choose to rely on LUS with or without integration with radiology methods. During that period, the imaging technique of choice was LUS, then, in case of clinical doubt or technical problems, X- Rays were used to overcome the issue.

At the bedside, anterior-posterior chest radiography was performed in the supine position following standard technique using a portable X-ray unit; radiologists were responsible for reading and interpreting the digital imaging. In our ICU during the selected period (2012–2014), three trained critical care physicians performed LUS. The operators had acquired the level of competence defined by the American College of Chest Physicians [12]. Trained physicians had performed theoretical courses, simulated practice on manikins (at least 50 scans) and had subsequent formal supervised practice [13]. The examination consisted of a bilateral scan of the anterior and lateral chest wall with patients in the supine position. A microconvex probe was used as the first choice for LUS. Then, to help resolve cases where diagnostic doubt remains, higher frequency probes were chosen for a better visualisation of the pleural line and subpleural space. The probe was positioned longitudinally in order to visualise the “bat sign” (the pleural line and two ribs), then, placed in transversal plane. Chest wall was examined in 8 areas with one scan for each area [14]. All regions were scanned with ultrasound in order to assess pleural sliding, presence and possible number of A- and B-lines, pleural effusion, pneumothorax, lung consolidation and diaphragmatic mobility. When indicated, two dorsal scans per side were added (consolidations, aeration monitoring) [15]. Ultrasound diagnoses of lung disease were defined according to the International Consensus Conference on Lung Ultrasound [16]. LUS findings were reported on the clinical notes.

For all patients we recorded sex, age, ICU-admission diagnosis (trauma, medical, surgical patients), SAPS II score, duration of ICU stay and ICU mortality. We recorded and compared the number of CRXs performed in Group A and Group B and the costs linked to CXR prescriptions between the two groups.

The data were entered into a spreadsheet (Microsoft Excel) and analyzed using SPSS (IBM SPSS software version 21). Demographic data are shown as means and standard deviation where appropriate. Analysis of data was performed using two-tailed Mann-Whitney test or Student t-test where appropriate: a value of P below 0.05 defined the significance. Cost for each medical exam was gathered from our regional price list.

## Results

A total of 4134 medical records were identified that fulfilled our inclusion criteria: 1869 patients in Group A and 2265 patients in Group B were included in our analysis. Demographic characteristics are shown in Table 1.

**Table 1:** Demographics and admission data of the participants: comparison between Groups A and B

Variables	Group A	Group B	P
Age (years)	61 [14.5]	63 [14.1]	0.79
Male (%)	52	56	0.14
Female (%)	48	44	0.12
SAPS II	20.2 [14.5]	20.6 [12.3]	0.28
ICU mortality	3.9	2.1	
ICU stay	2.74 [5.72]	2.85[8.06]	0.36
Admission diagnosis (%):			
Trauma patient	1	1	1
Surgical patient	86	89	0.9
Medical patient	13	10	0.9

Data are presented as mean and [standard deviation] or as percentage (%) where appropriate. SAPSII = The Simplified Acute Physiology Score II, ICU = Intensive care unit

**Fig. 1:**
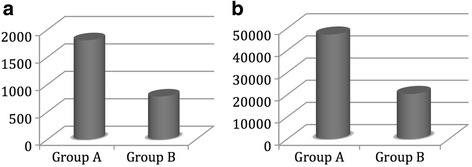
Clustered cylinder chart illustrates the differences in number (**a**, left chart) and cost (**b**, right chart) of chest X-rays between Group A and Group B. The Y-axis represents total number of chest X-rays (A, left chart) or the total cost in Euros (B, right chart)

Before the introduction of a routine use of LUS, we requested 1810 chest X-rays for diagnosis and follow-up (an average of 0.97 chest X-ray per patient) with an estimated cost of 47,060€. After the introduction of a routine use of LUS, we required 961 chest X-rays for diagnosis and follow-up (an average of 0.42 chest X-ray per patient) with an estimated cost of 24,986€. The significant differences in the total number of CXRs, average number per patient and estimated cost obtained before and after the introduction of routine application of LUS, are shown in Table 2. In Group B we observed a reduction in the number of CXR and relative cost by 57%, in comparison to Group A (Fig. 1). No statistically significant difference was observed in SAPS II regarding duration of ICU stay and ICU mortality between the two groups.

**Table 2:** CXRs and Ct requested and relative costs: comparison between Groups A and B

	Group A	Group B	P
Number of CXRs	1.810	961	0.012
Number of CXRs per patient	0.97	0.42	
Cumulative estimated cost due to CXRs (€)	47.090	24.986	0.012
Number of CT	87	89	

Data are presented as total number. CXRs; chest X-rays

## Discussion

In this study, we evaluated the influence that introducing a routine daily use of LUS may have on the number of CXRs and relative costs, in a polyvalent ICU. We found that the implementation of daily LUS examination leads to a statistically significant reduction in the number of CXRs without affecting the outcome. The cost savings associated with the reduction in the number of CXR was also remarkable. We did not find a decrease in the number of chest CT scans between the groups. The overwhelming majority of our sample consisted of surgical patients requiring a brief postoperative intensive care monitoring. For short ICU stays, the implementation of LUS seems of high significance, because, conventionally, X-rays are often routinely performed just before the patients’ transfer.

Regarding demographic and admission data, we decided to compare the two groups mainly on the basis of SAPS II because this score is valuable in predicting risk of mortality and it provides a reliable and effective estimation of the overall clinical condition of the patients at admission [17]. Consequently, we decided not to present the specific admission diagnosis and we categorized patients only on the base of three diagnoses: trauma, surgical and medical patients.

LUS was shown to be essential in our modern ICU, since it is a safe, fast bedside technique that may replace CXR for many applications. Moreover, the decreased radiation exposure represents a considerable improvement in safety and patient care. The carcinogenic effects of X-rays are extensively documented. The mechanism of radiation damage arises from the interaction between high-energy X-rays and biological material leading to radiation-induced mutations (a phenomenon known as “*stochastic effect”)* and to radiation-induced cell death (known as “*deterministic effect”*) [6]. Stochastic effects occur without a specific threshold dose of radiation; consequently, the risk of developing cancer from radiation exposure is essentially stochastic. Moreover, physicians have to take into account the cumulative risk of radiation exposure and that different imaging techniques require different doses of radiation. For instance, one chest CT scan has an effective radiation dose of 8 mSv, equivalent to 400 CXRs [18]. Furthermore, doctors have to consider that the risk for each dose also depends on age (higher in children, because of rapid dividing cells and life expectancy) and gender (higher in women). Consequently, the responsible use of imaging tests is vital in clinical practice. Inappropriate application of X-ray imaging is socially and economically unjustifiable. At the bedside with critically ill patients, CXR also suffers from methodological limitations (i.e., supine anterior-posterior vs upright posterior-anterior views, non-collaborating patient vs patient controlling his breath, possibility of adjunctive lateral views). These aspects, along with the presence of other concomitant lung diseases and complexity of critically ill patients, contributes to poor-quality CXR imaging and the possibility of wrong interpretations [19].

In this scenario, bedside point-of-care LUS has led to important changes in clinical practice, as it allows clinicians to rapidly answer specific questions, to guide therapy and to assess the efficacy of ongoing interventions, with high diagnostic accuracy and specificity. In 2004 Lichtenstein et al. evaluated the diagnostic accuracy of auscultation, bedside CXR and LUS in comparison to thoracic CT in patients with ARDS. These Authors showed that bedside CXR and physical examination had a lower sensitivity, specificity and diagnostic accuracy than LUS in the diagnosis of some lung conditions (i.e., pleural effusions, interstitial syndrome, alveolar consolidation) [5]. With the progression of literature on the topic and affirmation of a concept that has recently become evidence-based, the use of point-of-care ultrasound in the ICU has increased dramatically. Lee et al. [20] conducted a retrospective study in order to estimate the expected change over years in the use of imaging tests. They found a decrease in second-level imaging tests (CT by 21% and MRI by 6%) together with an increase in the use of ultrasound by 18.9%, and as a direct consequence a decrease in medical charges, without affecting patient outcome. In 2015, Zieleskiewicz et al. [11] investigated retrospectively the effect of ultrasound chest imaging on the reduction of CXRs and medical costs. They observed a statistically significant decrease in the number of CXRs due to the introduction of ultrasound in their ICU, without affecting mortality. Indeed, they did not find a decrease in the number of chest CT scans. Furthermore, Peris et al. [21] published an observational control study on 376 intensive care patients. The patient cohort was divided into two groups using temporal criteria (before and after the introduction of routine LUS). The data showed a statistically significant decrease in the number of CXRs and CT scans performed after the implementation of LUS. The conclusion of these Authors was that LUS may be used as an alternative to thoracic radiology [8] without affecting patient outcome [11]. Moreover, LUS is a valuable tool for selecting patients who effectively need a more advanced imaging technology (i.e., CT scan, MRI), thus contributing to reducing the number of inappropriate ionizing imaging tests.

The well-known and repeatedly cited limitation of sonography is operator dependency. A correct ultrasonography examination is directly related to operator skill, training, and experience. However, a combination of increased medical knowledge, ultrasound proficiency and appropriate training of operators could reduce errors [12]. Moreover, LUS is mainly based on simple basic signs and in many studies was shown to be highly reproducible in the hands of operators with different skills and experience, although the importance of correct training is always emphasized. Definitely, the benefits of implementing LUS in the ICU usually outweigh the pitfalls, if sonography is performed by trained physicians [22].

Our study presents several limitations. First of all, due to the retrospective nature of the study, it was not possible to gather information about the clinical indications for performing an imaging technique or when CXR was requested on the basis of US findings. For the same reason, it was not possible to evaluate how many chest x-rays or LUS examinations were normal and how many were pathological in the two groups. Second, the use of CXR or US was at the physician’s discretion, which may vary depending on the individual’s sensitivity and consideration. Since LUS is a relative novelty and is still considered a possible alternative, it was not feasible to set up a standard protocol for comparison. For these reasons, the reader should consider that the measured decrease in the number of CXRs observed in our study may not necessarily correspond to other situations. A future study on the subject should be performed, applying fixed protocols based on the evidence-based superiority of LUS in some applications. Third, it was not possible to collect information on overall mortality but only on ICU mortality, as patients were not followed-up after discharge from the ICU.

## Conclusions

In conclusion, this study shows the importance and effectiveness of LUS in reducing the number of CXRs performed in an academic polyvalent ICU. Routine LUS application, even when only left to the discretion of the caring physician, allows decreasing the use of ionizing procedures as well as related biological and economic costs.

## Abbreviations

CT: Computed tomography
CXR: Chest radiography
ICU: intensive care unit
LUS: Lung ultrasound
MRI: Magnetic resonance imaging
SAPS II: Simplified Acute Physiology Score
